# A Research Agenda for Malaria Eradication: Diagnoses and Diagnostics

**DOI:** 10.1371/journal.pmed.1000396

**Published:** 2011-01-25

**Authors:** The malERA Consultative Group on Diagnoses and Diagnostics

## Abstract

Many of malaria's signs and symptoms are indistinguishable from those of other febrile diseases. Detection of the presence of *Plasmodium* parasites is essential, therefore, to guide case management. Improved diagnostic tools are required to enable targeted treatment of infected individuals. In addition, field-ready diagnostic tools for mass screening and surveillance that can detect asymptomatic infections of very low parasite densities are needed to monitor transmission reduction and ensure elimination. Antibody-based tests for infection and novel methods based on biomarkers need further development and validation, as do methods for the detection and treatment of *Plasmodium vivax.* Current rapid diagnostic tests targeting *P. vivax* are generally less effective than those targeting *Plasmodium falciparum*. Moreover, because current drugs for radical cure may cause serious side effects in patients with glucose-6-phosphate dehydrogenase (G6PD) deficiency, more information is needed on the distribution of G6PD-deficiency variants as well as tests to identify at-risk individuals. Finally, in an environment of very low or absent malaria transmission, sustaining interest in elimination and maintaining resources will become increasingly important. Thus, research is required into the context in which malaria diagnostic tests are used, into diagnostics for other febrile diseases, and into the integration of these tests into health systems.

## Abstract 2

The Malaria Eradication Research Agenda (malERA) Consultative Group on Diagnoses and Diagnostics outline a research and development agenda to provide the diagnosis and diagnostic tools required for malaria eradication.

**Summary Points**

- New and improved screening tools and strategies are required for detection and management of very low-density parasitemia in the field
- Improved quality control is required for rapid diagnostic tests (RDTs) and microscopy in the field, to ensure confidence in diagnosis for case management
- More sensitive tests are required for *Plasmodium vivax* for case management
- Field-ready glucose-6-phosphate dehydrogenase (G6PD) deficiency tests and strategies for use to allow safe use of drugs against *P. vivax* liver stages are needed
- New strategies to manage parasite-negative individuals are needed to justify the continued inclusion of malaria diagnostics in febrile disease management in very low transmission areas.

## Introduction

As malaria transmission declines across much of its range and the possibility of elimination (reduction of transmission to zero in a defined geographical area) is increasingly considered [1],[2], accurate diagnosis and case identification through the demonstration of malaria parasites in sick patients presenting to health workers (“passive case detection”) is ever more important. During case management in all settings, all symptomatic patients with demonstrated parasitemia should be considered to be malaria cases, and all parasitemic patients should be given definitive antimalarial treatment. Accurate diagnosis is essential both to target antimalarial drugs and to enable effective management of the frequently fatal nonmalarial febrile illnesses [3] that share signs and symptoms with malaria [4]–[13].

However, the very low levels of transmission now being attained in many countries present new challenges that will demand new diagnostic tools and strategies, in particular, a change from passive case detection to “active” case detection. That is, as the elimination agenda is increasingly followed [14], improvements in current field diagnostics (microscopy and rapid diagnostic tests [RDTs]) for case management and new diagnostics that can detect very low levels of *Plasmodium* in the blood of asymptomatic individuals (and, in the case of *P. vivax*, in the blood of symptomatic individuals) who may contribute to continuing malaria transmission [15]–[21] will become essential. Furthermore, novel strategies will be needed to incorporate these new and improved diagnostics into routine health service activities.

More specifically, to avoid onward transmission, elimination programs for malaria will increasingly need to focus on detecting the highest possible fraction of infections in the general population through active rather than passive case detection. This change of focus will be essential because *Plasmodium* infections can persist at low densities for different lengths of time with no significant symptoms [16],[22],[23], and, in the case of *P. vivax* and *Plasmodium ovale*, as a latent stage in the liver that is not directly detectable. The contributions of these unseen reservoirs to the maintenance of transmission will depend on the success of detection and management of new cases and the coverage of vector and other control measures in the area [24],[25]. Thus, the usefulness of active case detection will vary with the epidemiology and health resources in an area and is itself a subject requiring further research [26].

Countries with successful “sustained control,” (the reduction of malaria transmission to a locally acceptable and sustained level through intensive use of vector control and effective case management) [14], will also need to adjust their diagnostic strategies as transmission declines to low levels and as they consider elimination. Importantly, until eradication of malaria (the reduction of transmission to zero worldwide) is achieved (and diagnostics therefore no longer required), efforts to eliminate malaria will continue to require diagnostics strategies as reintroduction will remain possible.

**Figure 1:**
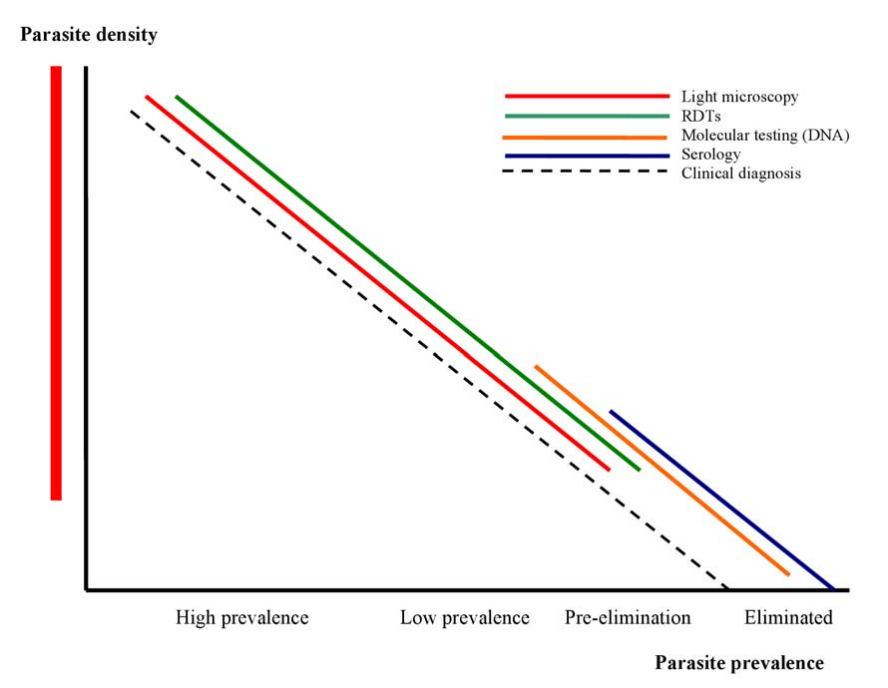
The position of different diagnostic approaches/tests in relation to morbidity, parasite prevalence, densities, and different stages towards malaria elimination. Image credit: Fusión Creativa.

This article, which summarizes the deliberations of the malERA Consultative Group on Diagnoses and Diagnostics, proposes a research agenda for the tools required for this process; related articles address broader issues of health service requirements and case management that will arise from their use [26],[27]. Figure 1 shows the position of different diagnostic approaches/tests in relation to morbidity, parasite prevalence, and densities and the different stages towards malaria elimination. Given the changing priorities for diagnoses and diagnostics as transmission reduces, in our discussion of the research needs for diagnostics, we distinguish between the two broad but overlapping areas of case management and surveillance/screening. This distinction is reflected in the target product profiles presented in Table 1. In both areas, sustainability will require integration with the general health system, and as much commonality as possible between diagnostics for different diseases. Thus we discuss priority setting in the context of the approaches already in use, or in the pipeline, for other diseases managed at the same levels of the health system. Because *P. falciparum* and *P. vivax* are the most prevalent plasmodia, the following discussion concentrates on these species, which most commonly present as mono-species infections. However, as *P. falciparum* infections decline, *P. ovale* may become relatively more prominent in areas where it is endemic, with implications for detection and management similar to those for *P. vivax*. Similarly, only time will tell whether transmission of *Plasmodium malariae*, which is transmitted across a broad geographical range, but at low prevalence, can be reduced using the measures applied to *P. falciparum,* or whether it will require specific strategies and tools. Notably, however, elimination of the zoonotic *Plasmodium knowlesi* is likely to require unique strategies (Figure 1).

**Table 1:** Target product profiles for malaria diagnostics.

Characteristic	Case Management in Elimination Settings	Screening/Surveillance (District Level or Below)
**Technical specifications**		
Analytic sensitivity (parasite/µl)^a^	E, 100–200, D<5^b^	E = 20, D≤5
Diagnostic sensitivity^a^	E>95%, D≥99%	E>95%, D≥99%
Analytic specificity	Negative all pathogens, common blood disorders	Negative all pathogens, common blood disorders
Diagnostic specificity	E>90%, D>95%	E>99% surveillance low-transmission areas, E>95% screening
Temperature stability	E>35°C, D>45°C^c^ (2 y)	E, 30°C; D, 45°C for short periods
Integrity of packaging	E, Moisture proof	E, Moisture proof
Species detection/differentiation:		
Pf predominant areas^d^	E, Pf; D, Pf/pan	E, Pf; D, Pf/pan
Pf and non-Pf areas	E, Pf/pan	E, Pf/pan; D, differentiation all species
Genotyping	No	No/O^e^
Ability to detect gametocytes	No	O
Ability to detect hypnozoites	No	D
**Health systems and technical specifications**		
Packaging of tests or reagents^f^	D, individual; D, all required consumables enclosed; D, bulk packaging displays temperature violations	D, all required consumables enclosed; D, bulk packaging displays temperature violations
Field stability/shelf life of consumables^g^	E, 2 y from manufacture (≥18 mo in country)	E, 12 mo (6 mo since country); D, 2 y from manufacture (≥18 mo in country)
Training requirements	D, half-day of community-level health worker	D, <1 wk of pretrained medical technician
Reagent requirements	E, nontoxic, all nonroutine provided; D, all necessary consumable items to perform the test provided in the kit	E, nontoxic, all nonroutine provided; D, all necessary consumable items to perform the test provided in the kit
Invasiveness	E, finger prick or less; D, noninvasive	E, finger prick or less; D, noninvasive
Rapidity of results^h^	E≤30 min; D≤15 min	E≤2 d; D≤half-day
Ease of use	Community: E, simple, few steps; Clinic: E, within medical tech ability; D, simple, few steps	E, within medical tech ability; D, simple, few steps
Cost	D≤US$1 per test	D≤US$1 per test
Safety	E, high blood safety with basic universal precautions	E, high blood safety with basic universal precautions
Waste disposal	Village-level waste disposal	Basic health system waste disposal
Inter-reader reliability (clarity of result)	Kappa>0.9	Kappa>0.9
Instrumentation and laboratory infrastructure requirements	E, no external power source; D, all provided with test	D, all provided with test

D, desirable; E, essential; O, optional.

a Analytic sensitivity*:* detection threshold against the marker of the infective agent (target) in controlled conditions. Diagnostic sensitivity*:* proportion (percent) of target cases detected by the test in the setting of intended use. The sensitivity required for *P. vivax* is generally at least that required for *P. falciparum*, and the parameters here should be applied to both. To achieve the required diagnostic sensitivity in low-prevalence settings, a greater analytic sensitivity (lower threshold of detection) may be required in some cases.

b Not required for febrile case management, but in an elimination setting, it would be desirable to detect incidental parasitemia at this level.

c Essential where stored in the field in ambient temperatures that frequently reach this level. Ambient temperature of prolonged storage in place of use should be considered the essential temperature stability requirement for a particular product.

d Areas in which infections are almost exclusively monospecies or mixed species *P. falciparum* infections. It is likely that many such infections have subpatent coinfections with other species. Where this represents a minority of infections, treatment on the basis of *P. falciparum* alone is likely to be acceptable from a programmatic and public health point of view. Non*-P. falciparum* infections are likely to become relatively more prominent as *P. falciparum* infections decline in prevalence, making the detection of non-*P. falciparum* species more desirable.

e May be of importance in areas undergoing certification for elimination.

f All inner (individual test) packaging should display, at a minimum: manufacturer name, product name, expiry date, lot number, target use (malaria).

g Outcome of temperature stability and integrity of packaging (ability to exclude moisture).

h Rapidity of results: For case management, results must be available before a patient is likely to leave the clinic. For surveillance, result availability in time for finding and managing cases is highly desirable.

## Diagnostic Strategies for Programs in the Intensified Control Phase

Identification of parasitemia in febrile patients is essential in all of the programmatic phases of the continuum from malaria control to elimination, although the challenges for health systems in maintaining this activity in areas where malaria has become rare will be more prominent, as will the importance of detecting asymptomatic infections of low parasite density. The ongoing role of other routine interventions, such as intermittent preventive treatment in pregnancy, needs reevaluating as elimination is approached. Moreover, because the distribution of malaria transmission is often highly heterogeneous within a country, strategies may need to vary at a subnational level. Analyses of past experiences and operations research are required to guide decisions on when these changes in emphasis should take place as control progresses [27],[28]. Although programs in areas of higher transmission will be less likely to engage in active case finding of individuals with low parasite densities, surveillance is nevertheless necessary to detect trends and the impact of interventions, and requires appropriate, high-throughput diagnostic tools. In addition to the diagnosis of malaria, it will be critical to have diagnostic capabilities for other causes of presenting illness, particularly fever. A sick adult or parent of a febrile child may not be satisfied with a diagnosis of “not malaria,” and both patients and providers require guidance on the integrated management of childhood illnesses, to ensure that appropriate alternative and specific treatment is available and provided.

Experience in eliminating malaria and maintaining elimination (or very low transmission) in sub-Saharan Africa is lacking, but experience from other areas suggests that resource requirements may be prohibitive and long-term maintenance of very low transmission and prevention of rebound unachievable using conventional management [29],[30]. Innovative approaches are therefore required. Diagnostic tools capable of detecting very low parasite densities (1 parasite/µl blood) in asymptomatic individuals will increasingly be required for active case detection and population surveillance to obtain a true picture of the prevalence of parasitemia and probability of transmission (as distinct from symptomatic malaria) [16]–[21]. Active case detection and treatment will be required whenever ongoing transmission is suspected and in high-risk populations (including those crossing borders), if the likelihood of ongoing transmission is to be eliminated. In these circumstances, test specificity is of increased importance because the absence of false positive results is critical in understanding the presence or absence of transmission [26].

## Diagnostic Strategies for Programs in Areas Where Elimination Has Taken Place

Once malaria is eliminated in a given area, considerable resources will be required to detect reintroduction through surveillance and to maintain capacity for rapid management and investigation of any cases found, as long as the risk factors that support transmission are still in place. Screening of migrant populations, screening of populations around detected cases, and case management tools for screening suspected patients, such as recent travelers or geographical associates of malaria cases may be needed. The tools to achieve these activities must be readily available in an environment where technicians are likely to be unskilled in the use of malaria diagnostic tests, particularly microscopy [27]. Thus, the requirements for surveillance and screening in areas where malaria has been eliminated, but risk of transmission is present, are similar to those of programs in an elimination phase. However, case management tools that are minimally dependent on previous technician experience in diagnosing malaria will be of particular importance.

## Diagnostic Tools for Case Management in an Elimination Setting

In settings where there is risk of autochthonous or imported malaria, diagnostics must be capable of rapidly and accurately detecting and quantifying parasitemia in febrile patients, and identifying species. In addition, highly sensitive diagnostic tools are needed for passive case detection and case management at health care facilities (public or private) that report to the national health information or disease surveillance systems. The issues around diagnostics in both case management and surveillance and control settings have a large impact on, and are impacted by, monitoring and evaluation requirements and health systems implementation issues such as the development of improved supply lines and logistics management, reporting of results and commodity consumption, and adherence of health workers and patients to management consistent with diagnostic results. These are all important areas where pooling of knowledge and sometimes operational research is required to maximize the impact of the diagnostic tools discussed below [26],[27].

## Light Microscopy

When performed to a high standard, light microscopy is capable of accurately identifying and quantifying *Plasmodium* parasites with sufficient rapidity for case management in most settings. It remains the operational gold standard in both control and elimination settings. However, the quality of light microscopy in the field is often inadequate [31]–[36] and limited by factors such as the instability and difficult preparation of currently used Romanowsky-based stains [37]–[39], poorly maintained, low quality equipment, and inadequate training, supervision, and quality assurance. Additionally, as malaria transmission decreases, it is likely that light microscopy technician skills may be redeployed elsewhere. Consequently, research into sustainable ways to maintain high-quality light microscopy in field settings, including innovative training, supervisory, and quality-assurance systems, is badly needed. More consistent and stable staining techniques are also required. This area of research has been ignored for the past 60 to 100 years, but has the potential to improve field accuracy significantly and may also improve the potential of the new reading techniques discussed below. Large volumes of slides pose particular challenges with respect to reading, especially in settings with low parasite prevalence where microscopist performance is hard to maintain [26].

## Digital Microscopy

Computer-assisted analysis of Giemsa-stained slides (possibly combined with automated staining), or digitized image transfer (potentially via mobile telephone) to a reference centre for review by an expert microscopist may enable greater consistency in parasite detection [40]–[44]. Additional research is required to determine whether these techniques will detect lower parasite densities than can be obtained by traditional light microscopy. Related techniques under development use software analysis of the scatter of various wavelengths of light to identify *Plasmodium* parasites and other pathogens. Although these digital techniques have the potential to improve field detection of malaria parasites, field-ready versions are not yet available, and it is not known whether these tools will meet the requirements for use in resource-poor settings.

## Fluorescent-Assisted Microscopy

Fluorescent-assisted microscopy (FAM)-based methods—for example, the quantitative buffy coat (QBC) method [45], incorporation of a fluorescent probe (fluorescence in situ hybridization [FISH]) or of parasite DNA [46], or antigen staining—has been used to a limited extent in various programs. FAM methods may eventually speed up slide reading and reduce operator error. High-throughput FAM may become possible if high specificity can be maintained by the absence of low artifactual staining. However, at present FAM cannot differentiate between species, a capability considered a major advantage of light microscopy over today's antigen-detection tests, although species-specific markers for FISH assays and fluorescent-tagged monoclonal antibodies are being developed. In addition, the applicability of FAM to parasite quantitation is not clear and FAM requires specialized equipment that will limit where it can be used.

## Antigen-Detecting RDTs

RDTs based on the detection of specific parasite antigens that use a platform design of lateral immunochromatographic flow (dipsticks or plastic cassettes) have started to change the way malaria is diagnosed in endemic settings. RDTs are increasingly being used at the community level and in control programs for case management and in prevalence surveys. Good RDTs reliably detect parasitemia down to 100–200 parasites/µl, which is comparable to the sensitivity of routine well-performed light microscopy [47]. In general, RDTs are simple to use. With training and quality assurance, they can be used by peripheral facility and village health workers to determine whether malaria parasites are present in a patient. However, increasing use in field settings suggests that many commercial RDTs have variable detection thresholds and field stability [48]. Systems for monitoring performance and routine quality control of manufactured product lots are therefore required.

Three parasite antigen types are targeted by currently available RDTs. Histidine-rich protein 2 (HRP2)-detecting tests have high sensitivity and specificity for *P. falciparum* but detectable antigen frequently persists after parasite clearance. The presence of HRP2 deletions in areas of South America also limits the use of these tests [49]. Commercial tests for *Plasmodium* lactate dehydrogenase (pLDH) have yielded variable results and, in general, have less potential to detect low parasite densities and greater susceptibility to deterioration under storage at high temperature than HRP2-based tests [48],[50]. However, species-specific (*P. falciparum* and *P. vivax*) and pan *Plasmodium* species-specific pLDH-based tests are available. Finally, tests targeting pan-specific parasite aldolase have shown inadequate detection thresholds in recent comparative trials, possibly because of the low concentrations of this target antigen in parasites [48].

The development of RDTs targeting other antigens may improve species identification (critical for elimination of *P. vivax*) and address some of the deficiencies of the current RDTs. In particular, current tests for *P. vivax*, which lack consistency in sensitivity and stability, might benefit from the use of monoclonal antibodies that target new antigens or improved manufacturing standards.

## Quality-Control Methods for Malaria RDTs

Standardized quality-control methods for RDTs are important for confirming test quality and ensuring that health workers and patients trust results. As with microscopy [39], quality assurance of RDTs requires a comprehensive, organized program [47],[51]. Such programs are absent in many countries. The development of standardized panels containing known concentrations of target antigens will greatly broaden the reach, applicability, and sustainability of RDT quality-control programs. Parasite-based panels that use cryo-preserved parasite preparations [52] are currently available at a centralized (regional) level, but panels that are easier to standardize and widely available are needed. Likewise, standardized regulatory approval and procurement in keeping with best practices will reduce the requirement for investment by individual procurement agencies in quality control and product evaluation programs. The development of low-cost tools for confirming quality at the national and field level (positive controls [53]) is also necessary to improve reach and sustainability. Finally, novel approaches that use PCR to confirm RDT results might eventually be useful.

## Diagnostic Tools for Active Case Detection and Community Surveys

For use in active surveillance and case finding, a diagnostic tool must be suitable for use in resource-poor field settings. Diagnostic tests must therefore be supportable at the district level or below, be affordable and low-maintenance, require less operator training than current methods, and have a low requirement for consumables. They should also detect very low parasite densities and distinguish between all locally prevalent *Plasmodium* species, be minimally invasive, and provide sufficiently rapid results to facilitate effective case management when an infection is identified. For use in prevalence surveys, where immediate management of asymptomatic parasitemia is not the aim, testing at a more centralized level may be sufficient. But, even in this context, rapid feedback and case management are desirable.

## Molecular (DNA) Detection

Current methods of detecting circulating parasites by demonstrating parasite DNA through amplification of ribosomal RNA (rRNA) genes by PCR assays represent the overall gold standard of malaria diagnostics. When sample concentration methods are used, 0.5 parasite/µl unconcentrated blood or lower can be detected. Quantitative PCR can be used to determine the concentration of circulating DNA and therefore estimate the density of circulating parasites. Survey and testing techniques, including pooling of samples, can reduce costs [54] but also reduce sensitivity to some extent by diluting samples.

At present, the application of PCR-based methods is restricted to well-equipped laboratories with specially trained technicians, partly because the need to avoid contamination (which leads to false-positive results) requires a very high standard of laboratory practice. PCR capacity is consequently limited in resource-poor malaria-endemic countries, where considerable investment would be required to establish and maintain it. PCR capacity-building programs are underway in several African countries through the Malaria Clinical Trials Alliance (MCTA). However, its restriction to well-equipped laboratories limits the applicability of PCR for surveillance and asymptomatic parasitemia case finding because timely feedback to allow the treatment of identified cases is impossible in most endemic areas. The development and field demonstration of high-throughput field-applicable PCR technologies is therefore needed to allow wider use of PCR in endemic settings.

Another molecular detection method based on DNA amplification is loop-attenuated isothermal amplification (LAMP). This method, which amplifies DNA (usually mitochondrial) with a single thermal cycle, has the potential to reduce the training and infrastructure requirements of molecular diagnosis [55]–[57], and would allow the timely feedback of results needed for case management. LAMP could also be used for surveillance, for detection of low-density parasitemia, and for monitoring parasite presence in antimalarial drug-efficacy monitoring and drug trials. However, LAMP has not yet been adequately field tested for wide-scale use or developed in a format suitable for the processes of high sample numbers.

## Hemozoin Detection

Hemozoin, a by-product of *Plasmodium* metabolism, can be detected through refraction/absorbance of laser light of certain frequencies, and has been used to detect malaria and to determine species. Current field-ready technologies are based on flow cytometers. Their application is limited to screening, however, because of low sensitivity at low parasite densities [58]–[62]. Current research activities include the development of transcutaneous hemozoin detection. If sufficiently sensitive and specific, this approach might offer a noninvasive test for malaria for mass-population screening of, for example, individuals moving into a malaria elimination area. Hemozoin detection may find a place in routine case management if appropriate tools can be developed.

## Antigen-Detection Tests

Current antigen-detecting RDTs (see earlier for details) are likely to miss a significant proportion of asymptomatic cases in low-transmission settings [16],[22],[23],[39]. Thus, although the current generation of RDTs can indicate the presence of malaria in a community, they cannot determine the true prevalence of parasite carriage. Research aimed towards increasing the sensitivity of existing RDTs may not change this situation because of the limitations of the currently available technology. Some antigen-detecting ELISAs are more sensitive than RDTs. Furthermore, because they can also be used to quantify antigen, they have been used to monitor drug efficacy. Antigen-detecting ELISAs may also facilitate high-throughput testing. However, their use is currently limited by laboratory and training requirements.

## Antibody Detection

Antibody detection (see also [27]) is currently available in ELISA and RDT formats, and is a sensitive way to demonstrate past exposure to malaria parasites (past infection). Because antibodies may not be detectable in blood-stage infections of very recent onset, these tests are inappropriate for case management. However, they may be useful in detecting established *P. falciparum* infections in which the blood-stage parasite density has fallen below the limits of light microscopy or antigen-detecting RDTs [63]. Detection of antisporozoite antibodies (so-called anti-CSP antibodies) alone or in combination with antibodies to blood-stage parasites has also been suggested as a surrogate for detecting individuals with a high likelihood of carrying *P. vivax* hypnozoites (evidence of infection) [64]–[68]. However, anti-CSP antibody responses are usually low and transient, especially in areas of low and moderate transmission, which renders this test unreliable.

Because antibody-detecting tests can identify parasite-infected individuals who are undetectable by antigen detection or light microscopy because of low parasite density, they could be used to screen populations such as migrants or blood donors to identify asymptomatic individuals at risk of transmitting malaria. They could also be used for identifying foci of recent transmission in areas that are otherwise malaria free and to determine the presence or absence of recent malaria transmission in specific populations, such as young children. They therefore have potential applications in confirming areas free of transmission during a defined period, provided they are further refined and developed in terms of sensitivity and specificity.

## Specific Issues for Reduction and Elimination of *P. vivax* Transmission

### Detection of Hypnozoites

*P. vivax* detection and management will become increasingly important as control measures reduce *P. falciparum* transmission. In many programs, *P. vivax* already causes the majority of clinical malaria episodes. Because *P. vivax* can remain latent in the liver but produces relapse, its effective management normally requires the use of 8-aminoquinolones to clear hypnozoites from the liver. No current diagnostic technique is capable of detecting *P. vivax* hypnozoites, and none are in development, although tests that can detect the presence of hypnozoites are a key research and development need wherever and whenever elimination has a chance of becoming a realistic goal. While symptomatic cases of *P. vivax* can be assumed to harbor liver stages and managed accordingly, a method for detecting hypnozoites would enable populations in *P. vivax*-endemic areas to be screened during the nontransmission season for asymptomatic individuals likely to have relapses who could then be treated before they become symptomatic and transmit in the following transmission season. Screening could therefore reduce the use of 8-aminoquinolones in mass-treatment programs in *P. vivax*-endemic areas, which would reduce the probability of drug-related severe side effects in glucose-6-phosphate dehydrogenase (G6PD)-deficient individuals (see next section). At present, compliance issues with the long course of primaquine (generally 14 days) have limited the broad application of this approach, and therefore the need for a diagnostic test for hypnozoites [24].

Potential biomarkers to detect hypnozoites include direct markers of metabolic activity, released antigens, markers of host immune response, and indirect serological markers of other stages (e.g., sporozoites). A lack of known markers of hypnozoite metabolic activity and markers of immunity limits the potential to assess the likely gains from investment in this area, and more knowledge of the biology of hypnozoites, perhaps through the development of liver-stage cultures, is required to determine whether such tests can be developed [69].

### Detection of G6PD Deficiency

The only drug currently licensed for the radical cure of *P. vivax* infection is primaquine, and the only investigational drug showing promise is tafenoquine, Both these 8-aminoquinolones cause hemolysis in G6PD-deficient individuals, the clinical importance of which varies with the particular G6PD-deficiency phenotype, and the starting hemoglobin concentration, and may depend on how the drugs are administered [70].

Because eliminating *P. vivax* reservoirs will probably involve the use of a hypnozoiticidal drug [24], unless a non–8-aminoquinolone drug is developed, G6PD testing is likely to be required for wide-scale elimination of *P. vivax*. The requirements for such a test differ somewhat from those of parasite-detecting RDTs, because testing should only be required once in a lifetime and is not urgently required; the use of hypnozoiticidal drugs can be delayed if necessary. So, for example, a G6PD test does not have the stability requirements of an antigen-detecting RDT. Current tests for G6PD deficiency nevertheless have limitations regarding storage requirements and the complexity of the procedure, so research is needed to develop new tests. Importantly, addressing G6PD deficiency will also involve research into test implementation—how should samples be tested, where should tests be done, and how should results be recorded to facilitate retrieval? Moreover, to decide whether further development of field-applicable G6PD tests is needed also requires more data on the distribution of G6PD phenotypes and on the efficacy and safety of alternatives to the standard hypnozoiticidal primaquine regimen.

## Other Research Priorities for Future Malaria Diagnostics

### Noninvasive Sampling

Current RDTs detect antigen in peripheral blood samples obtained by finger prick. This method is generally acceptable for case management in the formal health care sector, but it presents some logistical challenges at the community level and in some private sector settings, particularly with regard to the potential risks of blood-borne infection. In addition, invasive tests may not be fully accepted in some settings, particularly when taking samples from asymptomatic individuals, which could diminish access to malaria diagnosis, treatment, and surveillance. Noninvasive sampling (for example, saliva or urine collection) has the potential to overcome these impediments but, at present, the limitations of sensitivity of nonblood sampling are even greater than the limitations of blood sampling combined with antigen-detecting RDTs for screening and surveillance [71]–[73]. Published trials of antigen sampling from saliva and urine, for example, have demonstrated inadequate sensitivity, probably because of the low concentration of available antigen in these samples [71],[74]. Urine sampling may also present practical and cultural constraints. Techniques that concentrate antigen may have potential if they can be made practical for use in low-resource settings, but no such techniques are currently available. Additionally, if quantification is required, these methods would need to incorporate a standard to allow for variations in concentration of saliva or urine.

### Multiplexing

Multiple diagnoses from one assay or “multiplexing” is made possible by, for example, the inclusion of multiple PCR-based nucleic acid probes in a single test or the inclusion of antibodies specific for nonmalarial diseases or of pathological markers of disease severity. The inclusion of antibodies targeting nonmalarial diseases in RDTs in their common format (visually read immunochromatographic tests) increases the technical challenge of achieving the stability needed for sufficient shelf life and makes interpretation of results more complex. The usefulness of such tests is also limited by the ability of the health system to provide appropriate management for each etiological agent that may be identified, and the highly variable prevalence of potential target differential diagnoses within malaria-endemic areas.

However, as malaria rates drop through successful control programs, the overall fever rate may not change significantly. Accordingly, it will be increasingly important to integrate management of malaria with that of other febrile diseases, at the point of diagnosis, if the program is to remain credible and sustainable (see also [27]). Nonmalarial fever will need to be diagnosed with sufficient accuracy to allow practitioners to manage the main causes of fever successfully and to at least distinguish major bacterial infections manageable with common antibiotics from nonbacterial infections.

Research and development needs for multiplexing include the development of field-ready multiplex tests for malaria and nonmalarial diseases, which are not currently widely available, and research into the inclusion of markers for inflammation or severe disease in malaria tests, which would offer the potential to guide the referral of patients who require urgent management (see also [27]). Finally, the issue of complexity of interpretation in multidisease diagnostics needs to be addressed by the development of automated readers, particularly in combination with technology that allows multiple distinguishable markers to be captured in a single test line.

### Pooling Samples for Surveillance, Gametocyte Detection, and Genotyping

Three other potential research priorities were discussed by the Consultative Group, but the consensus was that research into pooling samples, gametocyte detection, and genotyping was less urgent. Thus, although the idea of pooling individual samples to detect parasitemia in very low transmission settings is intrinsically appealing and could result in cost savings using currently available tests, the Consultative Group felt that the limited quantity of antigen or DNA in pooled samples would severely limit the sensitivity of this approach. Similarly, the group decided that the development of a detection test for gametocytes should not be viewed as a high priority requirement. Finally, although WHO guidelines recommend genotyping of parasites during elimination phases [39], there is debate about whether research into methods for genotyping would be programmatically useful, particularly for *P. falciparum*. The resource needs to achieve genotyping are massive, and the long feedback time for results is likely to reduce the exercise to one of academic interest only. Genotyping could be useful for *P. vivax* infections to determine whether a blood-stage infection is new or a relapse. However, it has not yet been possible to develop methods that will reliably distinguish between relapse, recrudescence, and reinfection because of the multiplicity of hypnozoite genotypes present in *P. vivax*-infected individuals. Genotyping might, however, be useful in suspected outbreak or in new foci of transmission to determine the source of parasites, particularly when elimination in an area is being confirmed [26].

## Sustaining the Effort

The central importance of active case detection in each programmatic stage towards elimination has been comprehensively dealt with by several of the other malERA Consultative Groups [24]–[27]. However, whether active case detection can be achieved at sufficiently high and sustainable levels will depend to a great extent on the field utility and costs of the diagnostic and other tools eventually adopted for this role and on how these tests are used.

Importantly, when malaria is rare and no longer perceived by local health services and the community to be of significant public health concern, ways must be found to maintain the resources needed to test febrile cases for parasitemia to prevent resurgence of infection. Because malaria parasite detection will be competing for resources with other disease priorities with higher mortality, it will be necessary to target diagnostics to those cases more likely to be malaria rather than necessarily screening whole populations (although some form of screening, and the ability to respond rapidly to reintroduction, will continue to be necessary [26]–[28]. It will also be important to integrate malaria detection more fully with other health service activities and, as nonmalarial causes of fever become predominant, it will be critical to provide appropriate diagnosis and management of alternative causes so that compliance is maintained through confidence in the ability of the health system to provide solutions to clinical problems.

## Conclusions

Malaria elimination in the most challenging settings will require improvements in point-of-care tests for case management, and the development of new tests capable of identifying very low parasite densities in asymptomatic individuals in field settings for mass screening and treatment. As a result of our discussions, we propose a research and development agenda for diagnoses and diagnostics that should stimulate and facilitate the development, validation, and use of such tests (see Box 1).

**Box 1. Summary of the Research and Development Agenda for Diagnosis and Diagnostics**

**Overarching questions**

- What proportion of effort should be directed to screening and surveillance versus early case detection at various time points in elimination? Question to be addressed by modeling and validated in different areas.
- Do we need microscopy for elimination, or can other tests replace it?

**Programmatic issues**

- Further data on thresholds of (i) parasite density likely to cause symptoms in low-transmission settings with variable or waning immunity, and (ii) transmission potential of cases with parasitemia below the threshold of microscopy and RDTs
- Diagnostic tests for nonmalarial febrile illness in malaria-endemic and malaria-elimination settings
- Distribution of severe G6PD variants

**Technical issues: case-management tools**

*High priority*

Stable tests for case management in low-training, low-technology settings with sensitivity sufficient for community-level case management, including:

- Antigen-detecting RDTs
  - Greater consistency in *P. falciparum* detection, particularly in the case of nonpersistent antigens
  - More sensitive and stable tests to detect non*-P. falciparum* parasites
  - Clarification of the programmatic/implementation requirements that will ensure good impact in the field
  - Standardized low-cost positive controls for antigen-detecting RDTs suitable for field use
  - Sustainable tools for quality control of RDTs at a country level.
- Further investigation of nonblood sampling to determine the potential for detecting recoverable antigen in these samples.
- More consistent, reliable staining methods for microscopy
- G6PD deficiency mapping and identification (if 8-amino-quinolones are to be used)

*Medium priority*

- Multiplexing: Other diseases, markers of severity
- Field G6PD detection (may be more important if tafenoquine approved), or raised priorities for *P. vivax* relapse prevention
- Tools to standardize and improve microscopy interpretation

*Low priority*

- Hypnozoite detection (becomes a high priority if feasibility can be demonstrated through further research on hypnozoite biology, identifying good biomarkers).

**Technical issues: surveillance tools**

*High priority*

- Field-applicable tools for detection of low-density parasitemia in a high-throughput manner, suitable for surveys and active detection of parasite carriage in time to allow management of positive cases
- Tools for minimally invasive, very rapid detection of low-density parasite infections suitable for screening of migrants/travelers

*Innovation with potential for major operational impact*

- Noninvasive, low-density parasite detection

**Low-hanging fruit with immediate application for elimination**

- High-throughput field molecular detection, capable of use at district level or below
- Positive control methods for RDTs

Because malaria generally occurs in low-resource settings, the profits likely to be made from malaria diagnostic development and manufacture, particularly in the face of low mortality, are limited. The current market place for malaria rapid tests is dominated by small to medium-sized manufacturers, who are unlikely to be able to make the major investments needed to address these priorities alone. Thus, the role of donor agencies and product development partnerships and research institutions in enabling research and development and in providing the expertise and field access necessary to shape products to meet program needs will be an essential element of diagnostics development. Critically strong and focused, mainly public-private, partnerships will need to built and nurtured.

## Abbreviations

FAM: fluorescent-assisted microscopy
G6PD: glucose-6-phosphate dehydrogenase
RDT: rapid diagnostic test
